# HIF-1α drives distinct aspects of hypoxia-induced glucose metabolism in intestinal epithelial cells

**DOI:** 10.1016/j.jbc.2026.113193

**Published:** 2026-05-25

**Authors:** Sarah J. Kierans, Ciarán Kennedy, Rory Turner, Darragh Flood, Emily DeMichele, Martina Wallace, Cormac T. Taylor

**Affiliations:** 1 UCD School of Medicine, University College Dublin, Belfield, Dublin, Ireland; 2 Conway Institute of Biomolecular and Biomedical Research, University College Dublin, Belfield, Dublin, Ireland; 3 UCD School of Biomolecular and Biomedical Science & UCD Diabetes Complications Research Centre, University College Dublin, Belfield, Dublin, Ireland; 4 UCD School of Agriculture and Food Science & UCD Diabetes Complications Research Centre, University College Dublin, Belfield, Dublin, Ireland; 5 Systems Biology Ireland, University College Dublin, Belfield, Dublin, Ireland

**Keywords:** metabolomics, glucose metabolism, glycolysis, HIF-1, hypoxia, TCA cycle

## Abstract

The hypoxia-inducible factor-1α (HIF-1α) has canonically been described as a primary regulator of glucose metabolism in hypoxic cells through transcriptional upregulation of all 10 glycolytic enzymes. Here, using ^13^C-glucose and ^13^C-glutamine tracing in intestinal epithelial cells with defined HIF1A genetic perturbations, we demonstrate that hypoxia-induced glycolysis can occur independently of HIF-1-driven transcription. While hypoxia modulates glucose-derived carbon flux into anabolic branches of glycolysis independent of HIF-1α, HIF-1α plays an important role in modulating glucose and glutamine utilization within the tricarboxylic acid cycle. These alterations in substrate utilization highlight the layered regulatory framework whereby HIF-1α regulates distinct aspects of glucose and glutamine metabolism in intestinal epithelial cells to impact the rate of intestinal epithelial cell growth and promote metabolic adaptation to hypoxia.

In eukaryotic cells, glucose metabolism and the subsequent release of energy in the form of ATP is mediated by two metabolic pathways; first, oxygen-independent glycolysis, within the cytoplasm, and subsequently, by oxygen-dependent oxidative phosphorylation (OXPHOS), within mitochondria. Glycolysis, the first step in glucose catabolism, is a series of nine, non-oxidative reactions which convert glucose into two, three-carbon pyruvate molecules. This breakdown of glucose yields a net gain of 2 ATP (equivalent to about 5% of the total energy potentially available) per molecule of glucose which enters the pathway. In adequately oxygenated cells and tissues, the pyruvate generated from glycolysis is metabolized by the tricarboxylic acid (TCA) cycle and the electron transport chain during OXPHOS to generate 19 times more ATP per molecule of glucose consumed than glycolysis alone.

Under anaerobic conditions, or in cells lacking mitochondria (*e.g.,* erythrocytes), glucose is metabolized by glycolysis alone. Under such conditions, the absence of OXPHOS results in a low yield of ATP, necessitating an increase in glycolysis to maintain bioenergeticsstab homeostasis. This hypoxia-dependent induction in glycolysis, termed the Pasteur effect (1), is an active process, relying first on alleviation of allosteric control of glycolytic enzymes by ATP, and subsequently by the hypoxia inducible factor (HIF)-1-dependent transcriptional upregulation of genes encoding glycolytic enzymes and glucose transporters to increase glycolytic activity (2, 3, 4). This transcriptional upregulation of glycolysis, alongside an increased conversion of glycolytic end product pyruvate into lactate (5), allows for an increased flux through the glycolytic pathway and serves to maintain sufficient levels of cellular ATP production in hypoxia in the absence of OXPHOS.

While this adaptive response to hypoxia sustains energy production and supports cell survival during a hypoxic insult, cancerous cells can exploit this response to predominately rely on an enhanced rate of glycolysis for their growth and survival, irrespective of oxygen availability. This phenomenon, known as the Warburg effect (6), has significant consequences for tumor growth and survival, making understanding the complete mechanisms central to mediating this metabolic reprogramming of importance from both physiological and pathophysiological standpoints.

While earlier studies have defined a key role for HIF-1α in coordinating the metabolic response to hypoxia through transcriptional upregulation of glycolytic genes, more recently, we and others have described an induction of glycolysis in hypoxic cells independent of HIF-1-driven transcription (7, 8, 9, 10, 11, 12, 13), suggesting that additional mechanisms can regulate the Pasteur and Warburg effects. Here, using complementary, genetically modified, *in vitro* models, we show that homozygous deletion of HIF-1α in intestinal epithelial cells alters the mitochondrial metabolism of both glucose and glutamine, but surprisingly, does not affect the induction of glycolysis in hypoxic intestinal epithelial cells. We propose that HIF-1-mediated transcription and consequent glycolytic enzyme abundance is not the immediate rate-limiting factor for hypoxia-induced glycolysis. Furthermore, we propose that HIF-1 plays a more nuanced role in the control of glucose metabolism through the regulation of glycolytic branches and the TCA cycle.

## Experimental procedures

### Cell culture

For non-tracer studies, Caco-2 intestinal epithelial cells (ATCC, HTB-37) were cultured in DMEM containing 4.5 g/L glucose, 2 mM L-glutamine, 10% heat-inactivated foetal bovine serum, 1% MEM non-essential amino acids, and 1% penicillin–streptomycin (100 U/ml) and maintained in a humidified atmosphere of ambient air, supplemented with 5% CO_2_ and maintained at 37 °C. For hypoxic exposures, cells were incubated in pre-equilibrated tracer/control media at 1% O_2_, 5% CO_2_ and maintained at 37 °C in a humidified hypoxia chamber (Ruskinn, InvivO2 500). To assess cell growth, cell counts were carried out at time 0 (24 h post seeding immediately prior to hypoxic), 24 h and 48 h post-hypoxic exposure using a hemocytometer.

### 13C tracer studies

For isotopic labeling experiments, cells were cultured in 12-well plates in glucose- and glutamine-free DMEM (Gibco #A14430), containing 10% foetal bovine serum and 1% penicillin–streptomycin (100 U/ml) for 24 h. Media was supplemented with 25 mM unlabeled D-glucose (Gibco #A2494001) and [U-^13^C_5_] glutamine (Sigma #605166) or [U-^13^C_6_] glucose (Sigma #389374) and 2 mM unlabeled L-glutamine (Gibco #25030024). For all labeling experiments, media was pre-equilibrated to 1% O_2_, 5% CO_2_ and 37 °C in a humidified hypoxia chamber (Ruskinn, InvivO2 500) for 18 h prior to addition to cells.

### CRISPR-Cas9 editing

For HIF1A-/- cells, a guide RNA for HIF1A was designed using Benchling (www.benchling.com) and verified using the IDT CRISPR-Cas9 guide RNA design checker (https://www.idtdna.com/site/order/designtool/index/CRISPR_SEQUENCE) and the Synthego gRNA guide verification tool (https://design.synthego.com/) to identify a guide with consistently high on-target activity, low off-target efficiency and targeting as early an exon as possible. The chosen guide RNA for HIF1A, targeting exon 2 (5′-CCATCAGCTATTTGCGTGTG-3′), was cloned into the pSpCas9(BB)-2A-Puro (PX459) V2.0 construct, according to the protocol from Ran *et al.* (https://doi.org/10.1038/nprot.2013.143). Plasmid PX459 was a gift from Prof. Feng Zhang (Addgene plasmid #62988).

The PX459-sgRNA-HIF1A plasmid was transfected into cells using Lipofectamine 2000 (Invitrogen) and cells were selected using Puromycin (2 μg/μl). Clones were picked, expanded, and DNA was isolated for sequencing. Indel(s) were verified using sequence trace decomposition tool (https://academic.oup.com/nar/article/42/22/e168/2411890) accessed at https://apps.datacurators.nl/tide/.

The generation and validation of the HIF1A^NT^ cell line has been described previously by our group (10).

### HRE-*Gaussia* Luciferase reporter assay

HIF-1α functional activity levels were determined using a HRE-*Gaussia* Luciferase reporter system. Caco-2 cells were transfected with 750 ng of the HRE-*Gaussia* Luciferase reporter construct for 24 h in antibiotic-free media prior to their exposure to hypoxia (8–48 h). Secreted bioluminescence, indicative of HRE activity, was quantified using the Pierce Gaussia Luciferase Glow Assay Kit (Thermo Fisher Scientific, 16161). Relative Luminescence Units are presented normalized to total protein content (μg) for comparison of apparent HIF-1 functional activity.

### Immunoblotting

Whole-cell protein lysates were generated by lysing cells in lysis buffer (100 mM Tris-HCl, 300 mM NaCl, 10 mM MgCl_2_, 1% Triton X-100 supplemented with protease inhibitors (Sigma, P2714)). Isolated proteins were denatured, reduced, separated by SDS-PAGE and transferred to a 0.45 μm nitrocellulose membrane. Primary antibodies against HIF-1α (CST #36169), HIF-2α (CST #71565), hexokinase 2 (HK2;Invitrogen, MA525679), lactate dehydrogenase A (LDHA;CST #3582), glucose transporter subunit 1 (GLUT1;CST #73015), pyruvate dehydrogenase kinase 1 (PDK1;CST #3820), and β-actin (Sigma, A5441) were used alongside species-specific HRP-conjugated secondary antibodies [CST #7074 (Anti-Rabbit IgG), #7076 (Anti-Mouse IgG)] to probe for proteins of interest. The resulting signal was detected using enhanced chemiluminescence (Thermo Fisher Scientific, 32106) and imaged using the Vilber Fusion FX Chemiluminescent Imager.

### Endpoint metabolite analyses

Lactate, glucose, and glutamine were quantified in cell culture media using a CheKine Micro Lactate Assay Kit (Abbkine, #KTB1100), Glucose Colorimetric Detection Kit (Invitrogen, EIAGLUC), and EnzyChrom Glutamine Assay Kit (BioAssay Systems, EGLN-100), respectively, each carried out according to manufacturer’s instructions. Where indicated, metabolite levels are presented normalized to protein content (μg) for comparison of apparent metabolic rates.

### Metabolite extraction, derivatization and GC-MS analysis

After 24 h exposure to 21% or 1% O_2_, media was collected from each well for endpoint metabolite analyses. Cells were washed with ice-cold 0.9% NaCl and quenched using 0.25 ml of ice-cold HPLC-grade methanol (Merck #34860). 0.1 ml of water containing internal standard Norvaline (Sigma, N7502) was added to each well and cells were scraped and collected into eppendorf safe lock tubes (Eppendorf, #30120086). This step was performed within the hypoxia chamber to avoid sample reoxygenation. 0.25 ml HPLC-grade chloroform (Sigma #366927) was added to each tube and samples were vortexed at 4°C for 5 min. Samples were then centrifuged at 14,000 rcf for 5 min (4 °C). The aqueous phase containing polar metabolites was transferred to a new tube and evaporated under nitrogen.

Dried polar metabolites were derivatized in 2% (w/v) of methoxyamine hydrochloride (Sigma #89803) in pyridine and incubated at 40 °C for 60 min. Samples were then silylated with N-tertbutyldimethylsilyl-N-methyltrifluoroacetamide with 1% tert-butyldimethylchlorosilane (REGIS Technologies, #1-270143-200) at 45 °C for 45 min. Polar derivatives were analyzed by GC-MS using a DB-35MS column (30m × 0.25 mm i.d. x 0.25 μm, Agilent J&W Scientific) installed in an Agilent 7890B gas chromatograph interfaced with an Agilent 5977A MS with an XTR ion source using the following temperature program: 100 °C initial, increase by 5 °C/min to 215 °C, increase by 10 °C/min to 320 °C and hold for 4 min. The percent isotopologue distribution of each polar metabolite was determined and corrected for natural abundance *via* in-house matlab scripts using algorithms adapted from Fernandez *et al.* (14) and mass fragments outlined in Cordes *et al.* (15).

### Statistical analyses

Data are shown as mean ± SD from a minimum of three biological replicates. Statistical significance was determined in GraphPad Prism (V10.1.1; www.graphpad.com) using a two-way ANOVA, followed by Holm–Sidak *post hoc* test, or a two-tailed Student’s *t* test where appropriate. Asterisks denote the level of significance such that ∗ *p* ≤ 0.05, ∗∗*p* ≤ 0.01, ∗∗∗*p* ≤ 0.001, ∗∗∗∗*p* ≤ 0.0001.

## Results

### Hypoxia increases glycolysis in intestinal epithelial cells in the absence of HIF-1α

We have previously demonstrated that hypoxia induces glycolysis independent of HIF-1-driven transcriptional activity (10), suggesting that metabolic adaptation to hypoxia can occur through mechanisms beyond canonical HIF-1-mediated gene transcription. To further dissect the contributions of HIF-1α to the metabolic response to hypoxia, we used [U-^13^C_6_]-glucose tracing to track the fate of glucose-derived carbons in three genetically defined cell models: WT Caco-2 cells, homozygous HIF1A KO Caco-2 cells (HIF1A^−/−^), and HIF1A transcriptionally null Caco-2 cells (HIF1A^NT^), which express stabilized HIF-1α under hypoxia, but are unable to activate HIF-1 transcriptional targets.

**Figure 1:**
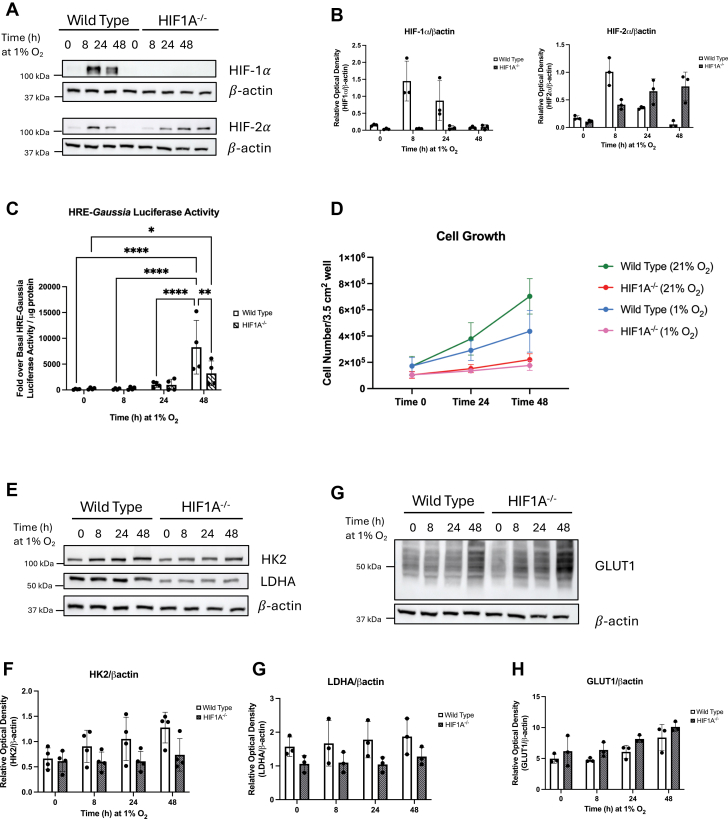
**Functional consequences of HIF1A knockout in intestinal epithelial cells.***A*, representative immunoblots of HIF-1α and HIF-2α stabilization in WT and HIF1A^−/−^ Caco-2 cells exposed to 21% or 1% O_2_ for 8 to 48 h. *B*, densitometric analysis of (*A*) relative to loading control, β-actin; n = 3 independent experiments. *C*, quantification of HIF-1α functional activity in WT and HIF1A^−/−^ cells transfected with a HRE-Gaussia luciferase reporter construct prior to their exposure to 21% or 1% O_2_ for 8 to 48 h. Results are shown as relative luminescence units over basal (21% O_2_) Relative Luminescence Units. Data are shown as mean ± SD for 3 independent experiments. *D*, quantitative analysis of cell growth following to 21% or 1% O_2_ for 0 to 48 h. Cell counts are expressed as mean ± SD of two technical replicates for each of the n = 4 independent experiments. *E* and *G*, representative immunoblots of HK2, LDHA, and GLUT1 in WT and HIF1A*−/−* cells exposed to 21% or 1% O_2_ for 8 to 48 h. *F* and *H*, densitometric analysis for (*E* and *F*) relative to loading control, β-actin; n = 3 independent experiments. β-actin controls shown in panel *A* are repeated in *panelsE* and *G* as these samples and were run on and probed from the same immunoblot. Statistical analyses were performed using a two-way ANOVA, followed by Holm-Sidak *post hoc* test. ∗ *p* < 0.05, ∗∗ *p* < 0.01, and ∗∗∗ *p* < 0.001.

Consistent with our previous findings, hypoxia increased intracellular and extracellular lactate accumulation (Fig. S1, *A* and *B*), in WT, empty vector control, and HIF1A^NT^ cells. Additionally, all genotypes displayed a similar enrichment of lactate from ^13^C glucose in hypoxia (Fig. S1, *C* and *D*), further indicating that in hypoxia, glucose metabolism can be reprogrammed toward lactate production in the absence of HIF-1-driven transcription. To evaluate how complete loss of HIF-1α affects the induction of glycolysis, we generated a single-cell derived HIF1A^−/−^ cell line using a CRISPR/Cas9 approach. Knockout of the HIF1A gene was confirmed by Sanger sequencing (Fig. S2), validated at the protein level by loss of hypoxia-inducible HIF-1α expression (Fig. 1, *A* and *B*), and functionally confirmed by a significant reduction in HRE-driven luciferase activity in hypoxia (Fig. 1*C*). While HIF1A knockout was successful, HIF-2α was stabilized in a hypoxia-sensitive, and time-dependent manner (Fig. 1, *A* and *B*), supporting the specificity and selectivity of our knockdown approach.

**Figure 2:**
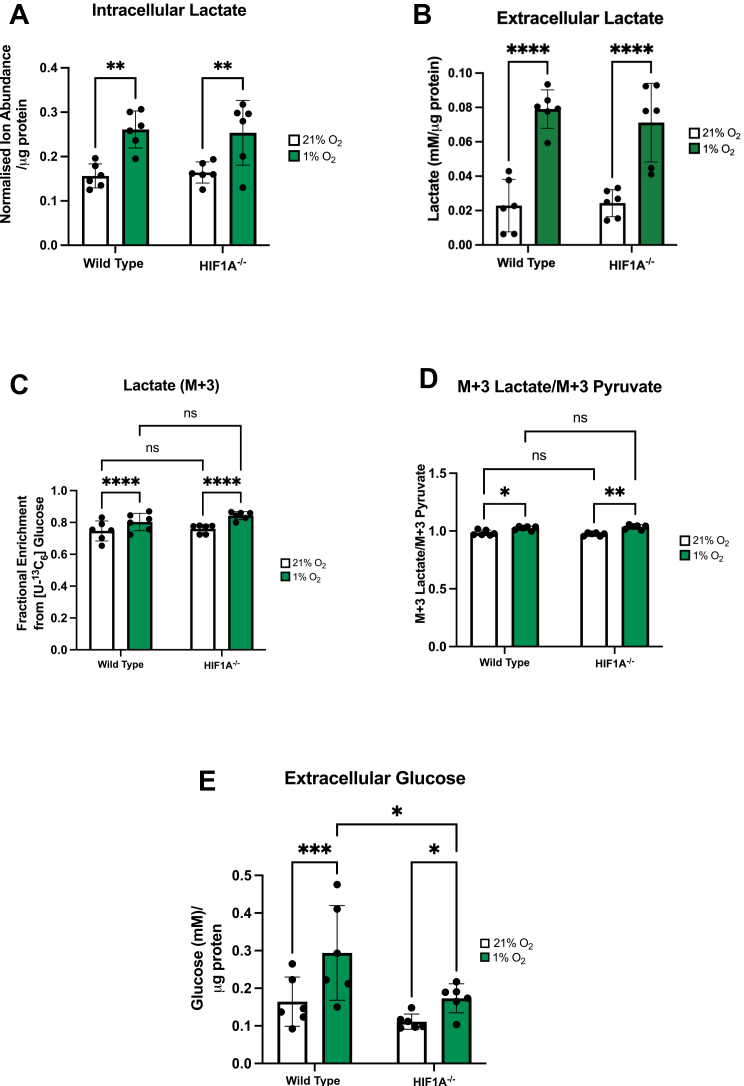
**Hypoxia induces lactate production independent of HIF-1a.***A* and *B*, intracellular and extracellular lactate accumulation from WT and HIF1A^−/−^ cells exposed to 21% or 1% O_2_ for 24 h. *C*, M + 3 labeling of lactate from [U-13C6] glucose in WT and HIF1A^−/−^ cells exposed to 21% or 1% O_2_ for 24 h. *D*, ratio of pyruvate to lactate labeling (M + 3 isotopomers) from [U-13C6] glucose in WT and HIF1A^−/−^ cells exposed to 21% or 1% O_2_ for 24 h. *E*, quantification of extracellular glucose following exposure of WT and HIF1A^−/−^ cells exposed to 21% or 1% O_2_ for 24 h. Data is presented as mean ± SD for n = 6 independent experiments. Statistical analyses were performed using a two-way ANOVA, followed by Holm-Sidak *post hoc* test. ∗ *p* < 0.05, ∗∗ *p* < 0.01, and ∗∗∗ *p* < 0.001.

We next assessed the functional consequences of complete HIF1A knockout. Under both normoxic and hypoxic conditions, HIF1A^−/−^ cells exhibited a lower rate of cell growth compared to WT counterparts (Fig. 1*D*). HIF1A^−/−^ cells showed an impaired induction of glycolytic enzyme, HK2, and oxidoreductase, LDHA (Fig. 1, *E* and *F*) in response to hypoxia, consistent with a decrease in HIF-1 dependent transcription. Interestingly, the hypoxia-inducible expression of glucose transporter GLUT1 was unaffected by HIF1A knockout (Fig. 1, *G* and *H*), suggesting redundancy in the HIF-1-dependent regulation of GLUT1 expression. Despite an impaired induction of glycolytic enzyme expression, HIF1A^−/−^ cells remained capable of increasing lactate production in hypoxia (Fig. 2, *A* and *B*). Additionally, both genotypes displayed a similar enrichment of lactate from ^13^C glucose (Fig. 2, *C* and *D*) indicating that in hypoxia, lactate production can be induced through mechanisms independent of HIF-1α and HIF-1-dependent gene expression. Interestingly, extracellular glucose levels were lower in HIF1A^−/−^ cells compared to WT cells following 24 h hypoxic exposure (Fig. 2*E*), indicating HIF1A^−/−^ cells rely more on glucose uptake than WT counterparts. However, extracellular glucose levels were higher in hypoxia than normoxia in both cell types suggesting that these cells do not increase glucose uptake in response to hypoxia.

### Hypoxia suppresses glucose-derived carbon flux into anabolic branches of glycolysis

**Figure 3:**
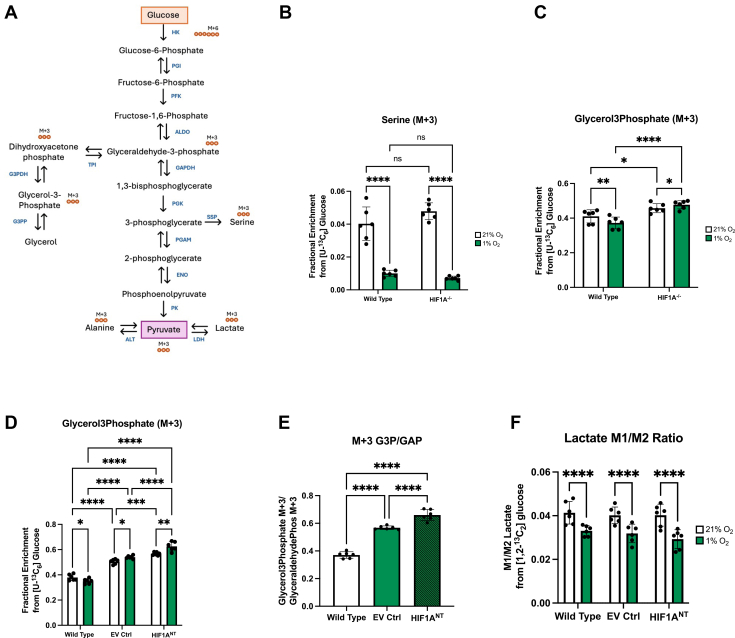
**Hypoxia alters glucose-derived carbon entry into anabolic branches of glycolysis via HIF-1a-dependent and -independent mechanisms.***A*, schematic of isotopomer distribution from [U-13C6] glucose incorporation into glycolytic metabolism and branching anabolic pathways. *B* and *C*, M + 3 labeling from [U-13C6] glucose in serine (*B*) and glycerol-3-phosphate (*C*) in WT and HIF1A^−/−^ cells exposed to 21% or 1% O_2_ for 24 h. *D*, M + 3 labeling of glycerol-3-phosphate from [U-13C6] glucose in WT, empty vector control, and HIF1ANT cells exposed to 21% or 1% O_2_ for 24 h. *E*, ratio of glycerol-3-phosphate to glyceraldehyde phosphate labeling (M + 3 isotopomers from [U-13C6] glucose in WT, empty vector control, and HIF1ANT cells exposed to 21% or 1% O_2_ for 24 h. *F*, ratio of M + 1 lactate to M + 2 lactate labeling (using [1,2-13C2] glucose) in WT, empty vector control, and HIF1ANT cells exposed to 21% or 1% O_2_ for 24 h. Data is presented as mean ± SD for n = 6 independent experiments. Statistical analyses were performed using a two-way ANOVA, followed by Holm-Sidak *post hoc* test. ∗ *p* < 0.05, ∗∗ *p* < 0.01, and ∗∗∗ *p* < 0.001.

Having observed that glucose derived lactate production is enhanced in hypoxia independently of HIF-1α, we next investigated the effect of HIF1A knockout on the partitioning of glucose-derived carbons into anabolic branches of glycolysis (Fig. 3*A*). In both WT and HIF1A^−/−^ cells hypoxia reduced the enrichment of glucose into serine (M + 3) (derived from glycolytic intermediate 3-phosphoglycerate) (Fig. 3*B*), indicating that the hypoxia-induced reduction in glucose enrichment into serine occurs independent of HIF-1α. In WT cells, hypoxia also caused a subtle but significant reduction in the enrichment of glucose into glycerol-3-phosphate (M + 3) (derived from glycolytic intermediates dihydroxyacetone phosphate) (Fig. 3*C*). In contrast, hypoxia increased glucose enrichment into glycerol-3-phosphate (M + 3) in hypoxic HIF1A^−/−^ cells, suggesting that HIF-1α may actively suppress glucose flux into anabolic pathways such as glycerolipid biosynthesis. To determine if HIF-1α regulates glucose partitioning into glycerol-3-phosphate by transcriptional or non-transcriptional mechanisms, we analyzed glucose enrichment into anabolic branches of glycolysis using our transcriptionally null model (HIF1A^NT^). HIF1A^NT^ cells displayed a similar hypoxia-dependent increase in M + 3 labeling in glycerol-3-phosphate, in addition to an elevated M + 3 glycerol-3-phosphate to M + 3 glyceraldehyde phosphate ratio (in equilibrium with dihydroxyacetone phosphate) (Fig. 3, *D* and *E*). Together, these findings suggest a role for HIF-1α transcriptional activity in limiting the diversion of glycolytic carbon into glycerolipid synthesis during hypoxia.

To complement the [U-^13^C_6_]-glucose tracing, we performed [1,2-^13^C_2_]-glucose tracing to further resolve the hypoxia-induced changes in glucose carbon partitioning. This tracer allows for resolution of glucose utilization through glycolysis *versus* the oxidative pentose phosphate pathway, thus providing additional insight into how HIF-1α transcriptional activity influences glucose utilization in hypoxia. Notably, hypoxia decreased the M + 1/M + 2 ratios for lactate in control and HIF1A^NT^ cells (Fig. 3*F*). This shift implies that hypoxia, independent of HIF-1 transcriptional activity, reprograms glucose metabolism to favor direct glycolytic activity (producing M + 2 lactate), rather than shunting glucose through the oxidative branch of the PPP (producing M + 1 lactate).

We have previously shown that hypoxia promotes interactions between glycolytic enzymes, independent of HIF-1-driven transcription (10). To determine if HIF-1α plays a non-transcriptional role in mediating and/or maintaining these interactions, we co-immunoprecipitated rate-limiting glycolytic enzyme, PFKP, from HIF1A^−/−^ cell lysates exposed to 21% O_2_ or 1% O_2_. While the interaction between glycolytic enzymes PFKP and HK2 were maintained in the absence of HIF-1α (Fig. S3), the hypoxic inducibility of these interactions was diminished, indicating a role for HIF-1α in the upregulation of these interactions in hypoxia. Collectively, these data suggest that the canonical model of HIF-1-driven transcriptional increase in glycolytic enzyme abundance alone does not adequately explain the hypoxia-inducible reprogramming of glucose toward increased lactate production and additional mechanisms contribute to this response.

### Hypoxia regulates glucose-derived carbon entry to TCA cycle *via* HIF-1α-dependent and -independent mechanisms

**Figure 4:**
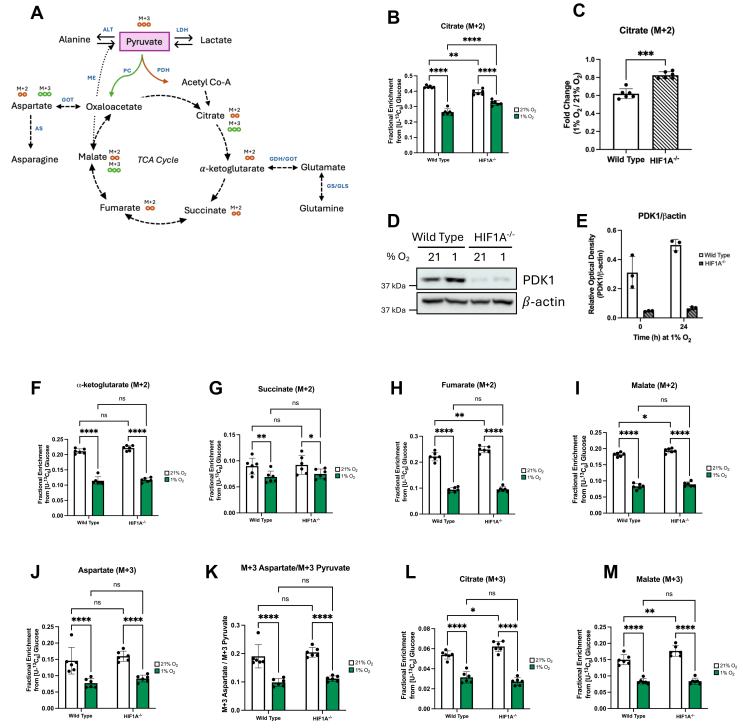
**Hypoxia alters glucose-derived carbon entry to the TCA cycle via HIF-1a-dependent and -independent mechanisms.***A*, schematic of isotopomer distributions from [U-^13^C_6_] glucose incorporation into the TCA cycle. The PDH complex-dependent oxidation of glucose-derived pyruvate to acetyl coA is indicated in *orange* (yielding M + 2 isotopomers of TCA cycle intermediates). The pyruvate carboxylase-dependent conversion of glucose-derived pyruvate to oxaloacetate (anaplerosis) is indicated in *green* (yielding M + 3 isotopomers of indicated TCA cycle intermediates). *B*, M + 2 labeling of Citrate and (*C*) fold change of (*B*) at 1% O_2_ reflecting relative incorporation of [U-^13^C_6_] glucose *via* PDH. *D*, representative immunoblots and (*E*) densitometric analysis of pyruvate dehydrogenase kinase 1 expression in WT and HIF1A^−/−^ Caco-2 cells exposed to 21% or 1% O_2_ for 24 h. (*F* − *I*) M + 2 labeling of TCA cycle intermediates, indicating incorporation of [U-^13^C_6_] glucose *via* PDH. *J*, *L*, *M*, M + 3 labeling of TCA cycle intermediates and (*K*) relative levels of glucose anaplerosis (aspartate (M + 3) to pyruvate (M + 3) labeling ratio) in WT and HIF1A^−/−^ cells exposed to 21% or 1% O_2_ for 24 h, indicating incorporation of [U-^13^C_6_] glucose *via* pyruvate carboxylase. Data is presented as mean ± SD for n = 6 independent experiments. Statistical analyses were performed using a two-way ANOVA, followed by Holm-Sidak *post hoc* test. ∗ *p* < 0.05, ∗∗ *p* < 0.01, and ∗∗∗ *p* < 0.001. PDH, pyruvate dehydrogenase; TCA, tricarboxylic acid.

Having demonstrated a hypoxia-dependent, but HIF-1α-independent induction of lactate production, we next investigated the effect of HIF1A knockout on glucose-derived carbon entry to the TCA cycle. Under hypoxic conditions, HIF-1α has been shown to suppress mitochondrial glucose oxidation by suppressing pyruvate dehydrogenase (PDH) activity, thereby limiting the conversion of pyruvate to acetyl co-A (*via* the PDH complex, Fig. 4*A*, orange arrow). In WT cells, hypoxic exposure reduced M + 2 isotopomer enrichment from glucose in citrate (arising from acetyl-coA (2C) and unlabeled oxalacetate (4C)) (Fig. 4*B*), as well as reducing M + 2 isotopomer enrichment in α-ketoglutarate, succinate, fumarate, and malate (Fig. 4, *F*–*I*). This reduction in M + 2 isotopomer enrichment is consistent with decreased PDH-mediated entry of pyruvate into the TCA cycle under hypoxic conditions. In contrast, HIF1A^−/−^ cells exhibited a more modest reduction in M + 2 citrate (Fig. 4, *B* and *C*) in hypoxia, highlighting that HIF-1α is required for full suppression of PDH activity in hypoxia. The HIF-1 dependency of PDH is further supported by a reduction in PDK1 expression in HIF1A^−/−^ cells (Fig. 4, *D* and *E*).

In addition to entering the TCA cycle *via* PDH, pyruvate can contribute to TCA cycle activity through anaplerosis, *i.e.* the conversion of pyruvate to oxaloacetate by pyruvate carboxylase (PC) (Fig. 4*A*, green arrow). In both WT and HIF1A^−/−^ cells, hypoxia reduced M + 3 aspartate labeling (derived from oxaloacetate) and decreased the M + 3 aspartate to M + 3 pyruvate ratio (Fig. 4, *J* and *K*), consistent with HIF-1-independent decrease in PC activity and anaplerosis from glucose-derived pyruvate. Hypoxia also reduced M + 3 enrichment in downstream metabolites, citrate and malate in both genotypes (Fig. 4, *L* and *M*), further supporting a HIF-1-independent downregulation of PC-mediated anaplerosis in hypoxia.

### HIF-1α promotes reductive carboxylation in hypoxia

**Figure 5:**
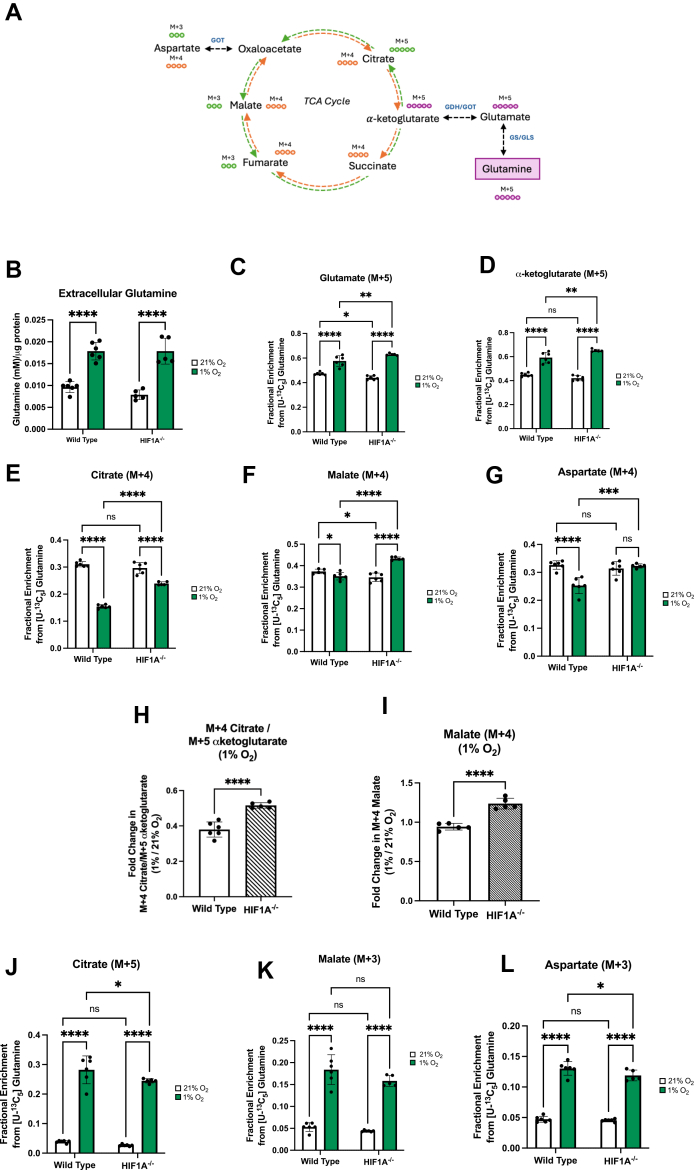
**HIF-1a promotes reductive carboxylation in hypoxia.***A*, schematic of isotopomer distributions from [U-13C5] glutamine incorporation into the TCA cycle. Reductive metabolism of glutamine-derived a-ketoglutarate (*green*) produces M + 5 citrate, M + 3 aspartate, and M + 3 malate. Oxidative metabolism (*orange*), yields M + 4 succinate, malate, aspartate, and citrate. *B*, quantification of extracellular glutamine following exposure of WT and HIF1A−/− cells exposed to 21% or 1% O_2_ for 24 h. *C* and *D*, M + 5 labeling of glutamate (*C*) and a-ketoglutarate (*D*) from [U-13C5] glutamine in WT and HIF1A−/− cells exposed to 21% or 1% O_2_ for 24 h. *E* and *G*, M + 4 labeling of citrate (*E*), malate (*F*), and aspartate (*G*), reflecting oxidative metabolism of [U-13C5] glutamine in WT and HIF1A−/− cells exposed to 21% or 1% O_2_ for 24 h. *H* and *I*, Ratio of M + 4 citrate to M + 5 a-ketoglutarate from [U-13C5] glutamine (*H*) and M + 4 malate (21% v. 1% O_2_, I) at 1% O_2_ indicating relative incorporate of [U-13C5] glutamine *via* oxidative metabolism. *J* and *L*, labelling of citrate (M + 5, *J*), malate (M + 3, *K*), and aspartate (M + 3, *L*), indicating incorporation of [U-13C5] glutamine *via* reductive carboxylation. Data are presented as mean ± SD for a minimum of n = 6 independent experiments. Statistical analyses were performed using a two-way ANOVA, followed by Holm-Sidak *post hoc* test, or two-tailed *t* test. ∗ *p* < 0.05, ∗∗ *p* < 0.01, and ∗∗∗ *p* < 0.001. TCA, tricarboxylic acid.

While glucose is an important substrate for energy production, glutamine is also an important carbon source for biosynthesis. To complement our [U-^13^C_6_]-glucose tracing, we performed [U-^13^C_5_]-glutamine tracing to assess the impact of HIF1A KO on glutamine utilization in hypoxia (Fig. 5*A*).

In both wild type and HIF1A^−/−^ cells, hypoxic exposure decreased glutamine uptake (Fig. 5*B*). However, the relative utilization of glutamine shifted toward a hypoxia-dependent, increased reliance on glutamine for the TCA cycle. For both genotypes, hypoxia increased labeling in M + 5 glutamate (Fig. 5*C*) and α-ketoglutarate in hypoxia (Fig. 5*D*), consistent with enhanced glutaminolysis and entry of glutamine-derived carbons to the TCA cycle in a HIF-1α-independent manner. In WT cells, hypoxia decreased M + 4 labeling of citrate, malate, and aspartate, indicative of a hypoxia-induced suppression of oxidative glutamine metabolism (Fig. 5, *E*–*G*). While oxidative glutamine metabolism was suppressed in hypoxic WT cells, hypoxia increased M + 5 labeling in citrate, M + 3 labeling in malate and aspartate (Fig. 5, *J* and *K*) indicative of a shift toward reductive carboxylation of glutamine-derived carbons in hypoxic WT cells. This shift toward reductive carboxylation was further supported by an increase in the α-ketoglutarate: citrate ratio (Fig. 5*M*); a primary driver of reductive carboxylation (16). In contrast, HIF1A^−/−^ cells retained a greater capacity for oxidative glutamine metabolism in hypoxia, as evidenced by an increased M + 4 labeling in malate (Fig. 5, *F* and *I*), a preserved M + 4 labeled in aspartate (Fig. 5*G*), and an increased M + 4 citrate to M + 5 α-ketoglutarate ratio (Fig. 5*H*) in hypoxia. These findings implicate HIF-1α in the hypoxia-induced suppression of oxidative glutamine metabolism and the redirection of glutamine flux toward reductive carboxylation.

### Redirection of glutamine metabolism requires HIF-transcriptional activity

**Figure 6:**
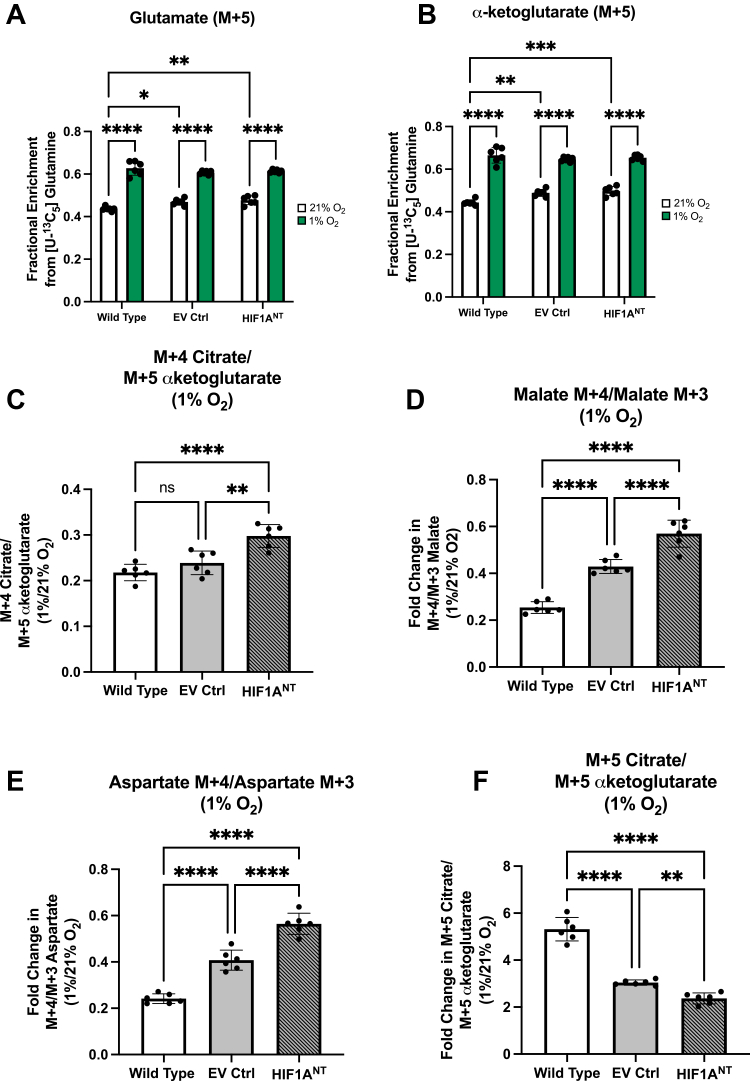
**Hypoxia-inducible redirection of glutamine metabolism requires HIF-1-driven transcription.** M + 5 labeling of glutamate (*A*) and a-ketoglutarate (*B*) from [U-13C5] glutamine in WT, empty vector control and HIF1ANT cells exposed to 21% or 1% O_2_ for 24 h. *C*–*F*, ratio of oxidative to reductive metabolism in WT, empty vector control and HIF1ANT cells exposed to 21% or 1% O_2_ for 24 h.

Finally, to determine whether the HIF-1α-dependent suppression of oxidative metabolism in hypoxia is related to HIF-driven transcription or an additional non-transcriptional role for HIF-1α, we assessed glutamine utilization in HIF1A^NT^ cells. WT, empty vector control, and HIF1A^NT^ cells all exhibited an increase in M + 5 labeling in glutamate (Fig. 6*A*) and α-ketoglutarate in hypoxia (Fig. 6*B*), confirming enhanced glutaminolysis and entry of glutamine-derived carbons to the TCA cycle, independent of HIF-1-driven transcription. When compared to WT cells and empty vector control cohorts, HIF1A^NT^ cells retained a greater capacity for oxidative glutamine metabolism, as evidenced by hypoxia-inducible increase in the M + 4 citrate to M + 5 α-ketoglutarate ratio at 1% O_2_ (Fig. 6*C*) and M + 4 to M + 3 ratios for both malate and aspartate (Fig. 6, *D* and *E*). This increased in the oxidative metabolism of glutamine was supported by a hypoxia inducible reduction in the M + 5 citrate to M + 5 α-ketoglutarate ratio in HIF1A^NT^ cells (Fig. 6*F*), indicative of a decreased reductive carboxylation of glutamine-derived α-ketoglutarate. These data suggest that redirection of glutamine metabolism toward reductive carboxylation in hypoxia requires intact HIF-1α transcriptional activity.

## Discussion

Using complementary genetic models – a homozygous HIF1A knockout (HIF1A^−/−^), a transcriptionally inactive HIF1A mutant (HIF1A^NT^), and WT cells, we show that intestinal epithelial cells retain their capacity to reprogram glucose metabolism in hypoxia in the absence of HIF-1α and HIF-1-dependent transcriptional upregulation of glycolytic enzymes expression. These challenges the canonical model of hypoxia induced glucose reprogramming in which HIF-1-driven glycolytic gene expression is a primary mechanism underpinning the increase in glycolysis in hypoxia, and suggests that enzyme abundance is not a rate-limiting factor for hypoxia induced glycolysis to proceed. This finding is supportive of our previous work, highlighting the involvement of additional non-transcriptional mechanisms in the induction of glycolysis in hypoxia (10), and work from others (7, 8, 9, 11, 12, 13) describing HIF-1-independent elevated glycolytic flux in hypoxia in multiple *in vitro* models. This study raised the possibility that contrary to work from others in the field (3), HIF-1α is redundant in the reprogramming of glucose metabolism toward lactate production in hypoxia, at least in intestinal epithelial cells. From an evolutionary perspective, the ability to maintain high glycolytic flux without HIF-1α is perhaps unsurprising, given that glycolysis predates multicellularity and oxygen-dependent life by approximately three billion years. This layered regulation of metabolism likely evolved to safeguard an essential bioenergetic function in fluctuating oxygen environments, enabling eukaryotic cells to rapidly increase their glycolytic capacity in a time frame incompatible with transcriptional regulation.

While we observed an induction of lactate production independent of HIF-1α, it is notable that in contrast to other cell types, hypoxia decreased total glucose uptake in both genotypes. Despite this reduction, relative glucose utilization shifted toward an increase diversion of glucose to lactate. This combination of decreased glucose uptake and increased lactate production may be explained by reduced channeling of glucose into anabolic branches of glycolysis, such as those involved in amino acid synthesis (*e.g.*, serine). This reprogramming would permit greater conversion of glucose into lactate and concomitantly reduce glucose entry into the TCA cycle without requiring an enhanced uptake of glucose.

With regards to a potential mechanism by which glucose metabolism is reprogrammed in hypoxia, independent of HIF-1-driven gene expression, it is noteworthy that while HIF1A knockout impaired the induction of glycolytic enzymes, HIF1A^−/−^ cells remained capable of upregulating glucose transporter, GLUT1, in a hypoxia-dependent manner. This suggests that maintaining glucose availability and intracellular flux, rather than increasing net uptake or glycolytic enzyme expression, may be sufficient to drive glycolysis through mass-action effects (17). Additionally, the induction of glycolysis, independent of HIF-driven transcription, may be further explained by the formation of glycolytic enzyme complexes, characterized in our previous work (10). In this study, these complexes were identified to interact basally in a HIF-1-independent manner, but the hypoxia induced enhancement of these interactions was lost in HIF1A^−/−^ cells. While it is probable that a reduction in enzyme abundance may contribute to the reduced level of interactions, we cannot discount a possible role for HIF-1α in regulating scaffolding formation or accessory protein expression that reinforce glycolytic complex organization during periods of prolonged hypoxic stress. The formation of these complexes may enhance pathway activity by facilitating channeling of substrates or indeed mass action, and help ensure that enzymes are not require to operate at maximal, and instead, provide reserve capacity for when required.

Although glycolysis can be induced in hypoxia in the absence of glycolytic gene induction, this work does not discount the role for HIF-1α in coordinating the metabolic response to hypoxia. While glucose metabolism is pertinent in the generation of cellular energy, intermediates generated from glycolysis and the TCA cycle also play important roles in promoting biomass synthesis and redox homeostasis. In both knockout and transcriptionally null models, hypoxia redirected glucose toward glycerol-3-phosphate, suggesting that HIF-1α normally suppresses glucose entry into this lipid biosynthetic branch in hypoxia. This diversion may reflect an adaptive strategy to preserve glucose within the glycolytic pathway for ATP production, and limit energetically costly lipid biosynthesis. However, other anabolic branches, such as serine biosynthesis and the pentose phosphate pathway, were reduced regardless of HIF-1α expression, suggesting additional oxygen-sensitive mechanisms regulate the provision of glucose derived carbons into these anabolic routes.

In addition to the enhancement of glycolysis, cellular adaptation to hypoxia also requires an active suppression of pyruvate catabolism *via* the TCA cycle. Our [U-^13^C_6_] glucose tracing was supportive of the role for HIF-1α in suppressing PDH-mediated pyruvate entry, a finding consistent with HIF-1-dependent induction of PDK1, which phosphorylates and inactivates the PDH complex to prevent pyruvate conversion to acetyl co-A (5, 18). However, in contrast to others, who have observed a HIF-1-dependent decrease in PC activity in hypoxia (19), we observed a HIF-independent decrease in pyruvate mediated anaplerosis in our model, suggesting both HIF-dependent and -independent mechanisms are responsible for reducing pyruvate entry to the TCA cycle.

Finally, while glucose is the primary substrate used for ATP production, glutamine is also an important source of carbon and nitrogen for the synthesis of nonessential amino acids aspartate, asparagine, glutamate and proline (20, 21). In this study, we demonstrate that hypoxia increases glutamine entry into the TCA cycle, in a HIF-1-independent manner, despite a reduced uptake of glutamine in both genotypes. It is possible that hypoxia reduces the use of glutamine for amino acid synthesis and other biomass-related pathways, thereby lowering total glutamine uptake. Nevertheless, glutamine flux into the TCA cycle can still be increased, as demonstrated here. Once within the TCA cycle, glutamine had contrasting metabolic fates depending on HIF-1α expression. Hypoxic WT cells shifted toward reductive glutamine metabolism, a finding consistent with other studies (16, 20, 21, 22, 23, 24, 25, 26, 27, 28). This metabolic switch toward enhanced glutaminolysis is presumably, in part, a protective mechanism to promote oxidative homeostasis and glutathione production for protection against oxidative stress (29, 30). Additionally, reductive glutamine metabolism is important for mediating lipogenesis *via* citrate in hypoxia (16, 23, 24, 28, 31). This switch from oxidative to reductive glutamine metabolism was significantly reduced in HIF1A^−/−^ cells, offering a potential explanation for the reduction in cellular proliferation observed in in both normoxic and hypoxic HIF1A^−/−^ cells. While speculative, the increased diversion of glutamine into lipogenic pathways may compensate for the HIF-dependent diversion of glucose-derived carbons away from glycerol-3-phosphate synthesis observed in this study. Future work will focus on addressing the implications for HIF1A^−/−^ on hypoxic *de novo* lipogenesis.

Collectively, these findings underscore the complexity of metabolic regulation in hypoxia and support a model in which HIF-1-mediated transcription and consequent glycolytic enzyme abundance is not the immediate rate-limiting factor for hypoxia-induced glycolysis. We propose that while the initial increase in glycolytic rate is driven primarily by HIF-1-independent mechanisms, HIF-1-driven transcription fine-tunes substrate fate and may provide reserve capacity for sustained energy production during periods of prolonged hypoxia. While this confers an adaptive advantage to cells deprived of an adequate oxygen supply, therapeutically, this redundancy suggests that targeting HIF-driven enzyme expression alone may have limited effect on glycolytic throughput in immune, endothelial, or tumor cells, unless the accompanying non-transcriptional mechanisms are also addressed.

## Data availability

All data generated or analyzed during this study are included in this article and the supplementary information files.

## Supporting information

This article contains supporting information.

## Conflict of interest

The authors declare that they have no conflicts of interest with the contents of this article.
