# Supplementary material for: HIF-1α drives distinct aspects of hypoxia-induced glucose metabolism in intestinal epithelial cells
Source: J Biol Chem. 2026 May 25;302(7):113193. doi: 10.1016/j.jbc.2026.113193 (PMC13314749; doi:10.1016/j.jbc.2026.113193)
Supplement: Supplemental Figures [file mmc1.pdf]

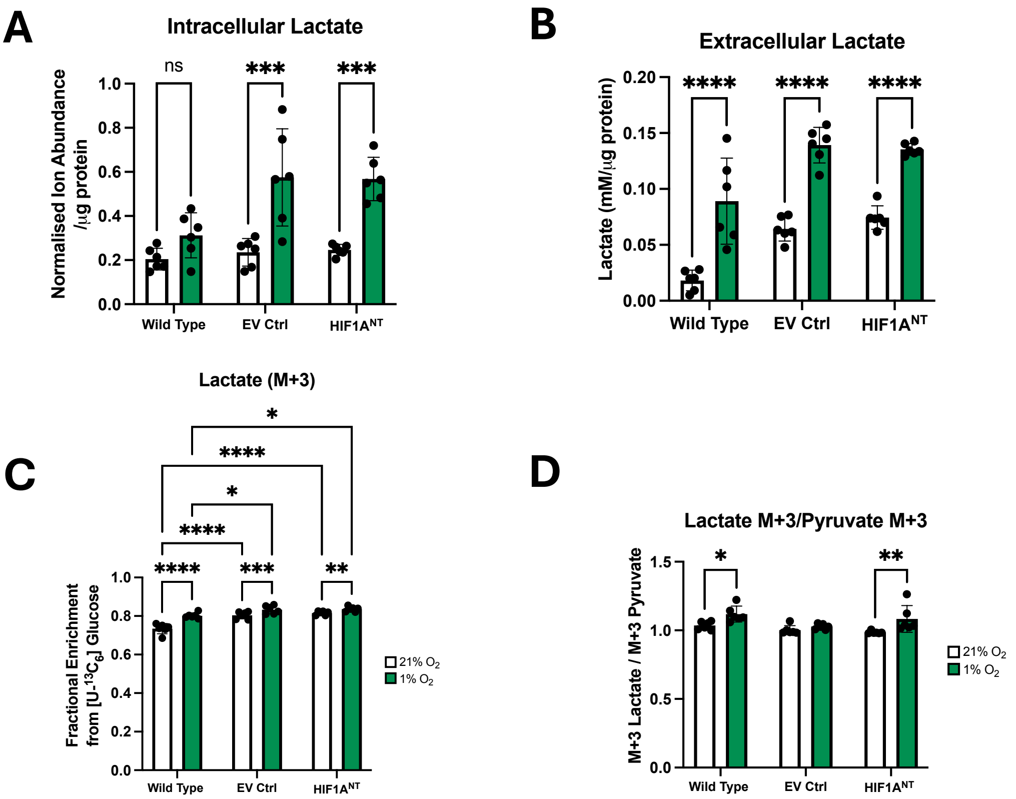

Supp. Fig. 1: (A) Intracellular and (B) extracellular lactate accumulation from wild type, empty vector control and HIF1A^{NT} cells exposed to 21% or 1% O_2 for 24 h. (C) M+3 labelling of lactate and (D) the ratio of lactate to pyruvate labelling (M+3 isotopomers) from [U-$^{13}C_6$] glucose in wild type, empty vector, and HIF1A^{NT} cells exposed to 21% or 1% O_2 for 24 h. Data is presented as mean ± SD for n = 6 independent experiments. Statistical analyses were performed using a two-way ANOVA, followed by Holm-Sidak post hoc test. ∗ $p < 0.05$, ∗∗ $p < 0.01$, and ∗∗∗ $p < 0.001$.

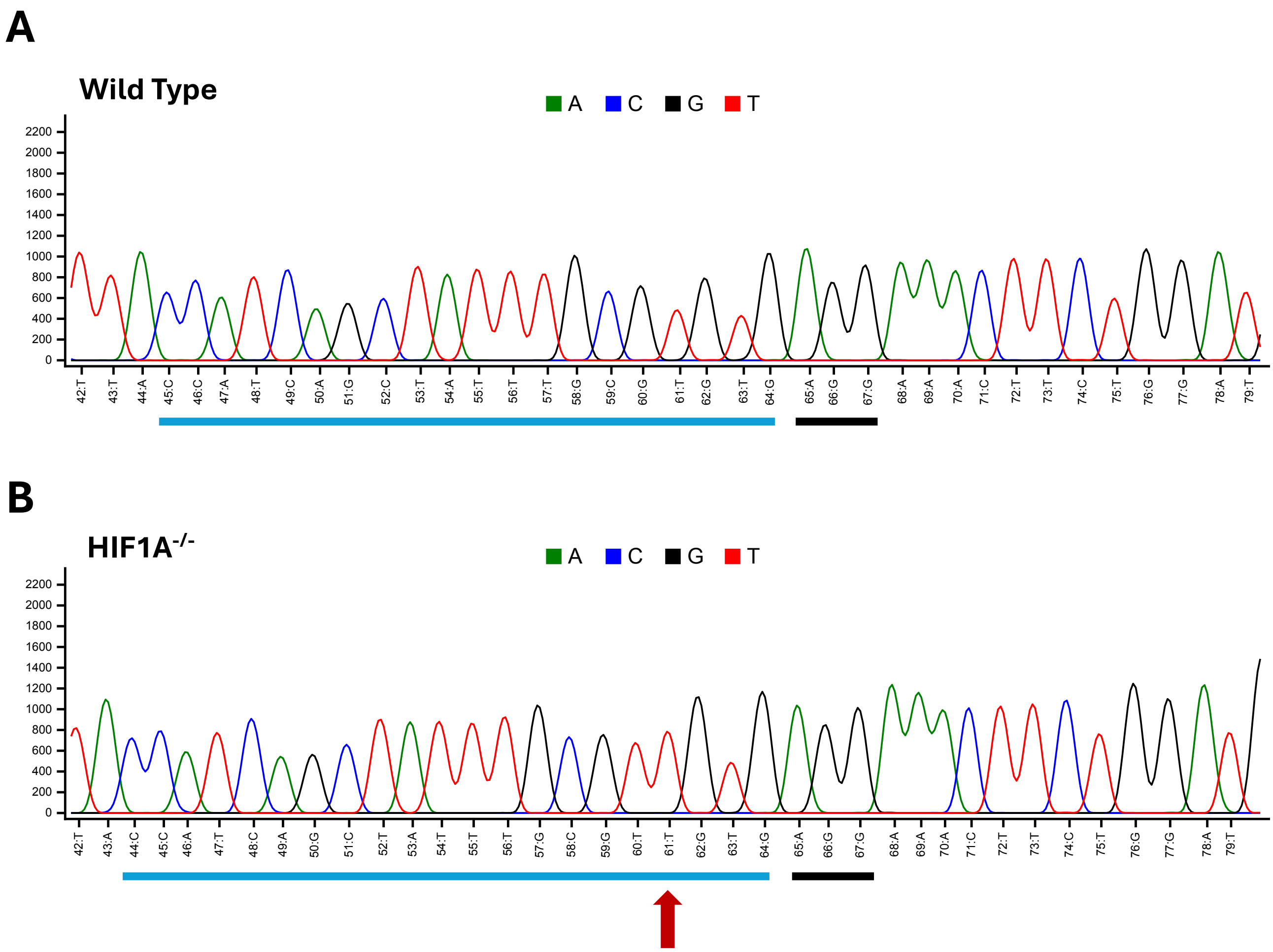

Supp. Fig. 2: Sanger sequencing results of (A) wild type and (B) HIF1A$^{-/-}$ cells. The PAM sequence is highlighted in black. Guide sequence highlighted in blue. The single-base insertion leading to a missense mutation in the HIF1A gene is indicated by the red arrow.

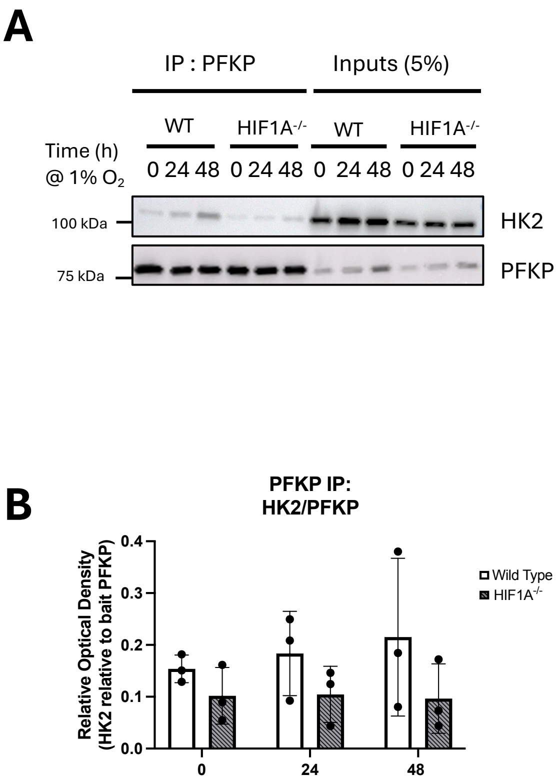

Supp. Figure 3: (A) Representative immunoblots reflecting HK2 and PFKP expression following co-IP of PFKP from whole cell lysates of wild type and HIF1A$^{-/-}$ cells exposed to 21% or 1% O_2 for 8 to 48 h (Input = 5% total IP). (B) Densitometric analysis of (A) relative to bait, PFKP. Data is presented as mean ± SD for n = 3 independent experiments.

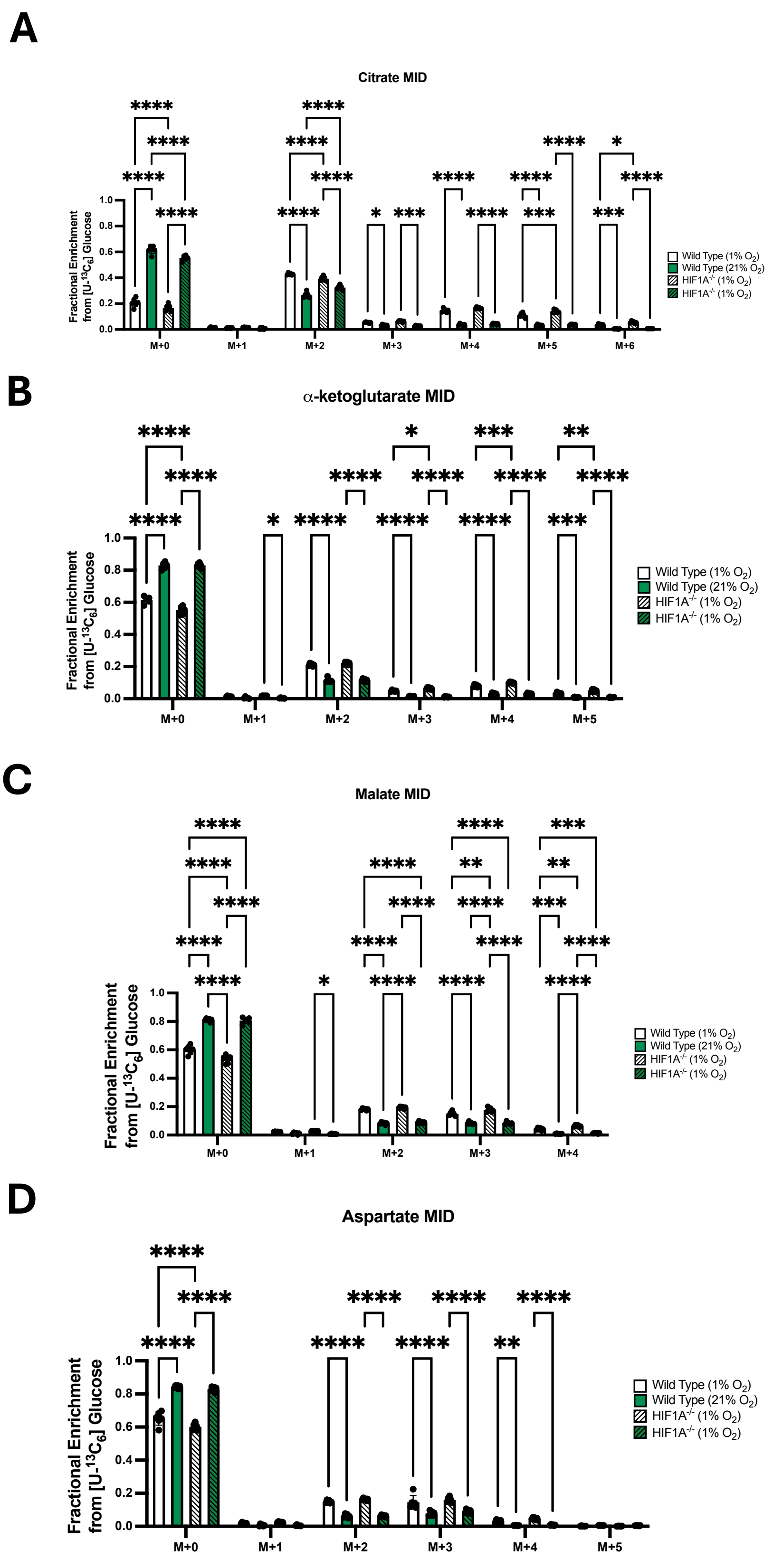

Supp. Figure 4: Isotopologues distributions for (A) citrate, (B) α-ketoglutarate, (C) malate, and (D) aspartate, from [U-$^{13}C_6$] glucose in wild type and HIF1A$^{-/-}$ cells under normoxic (21% O_2) and hypoxic (1% O_2) conditions (24 h). Data is presented as mean ± SD for n = 6 independent experiments. Statistical analyses were performed using a two-way ANOVA, followed by Holm-Sidak post hoc test. $*$ $p < 0.05$, $**$ $p < 0.01$, and $***$ $p < 0.001$.

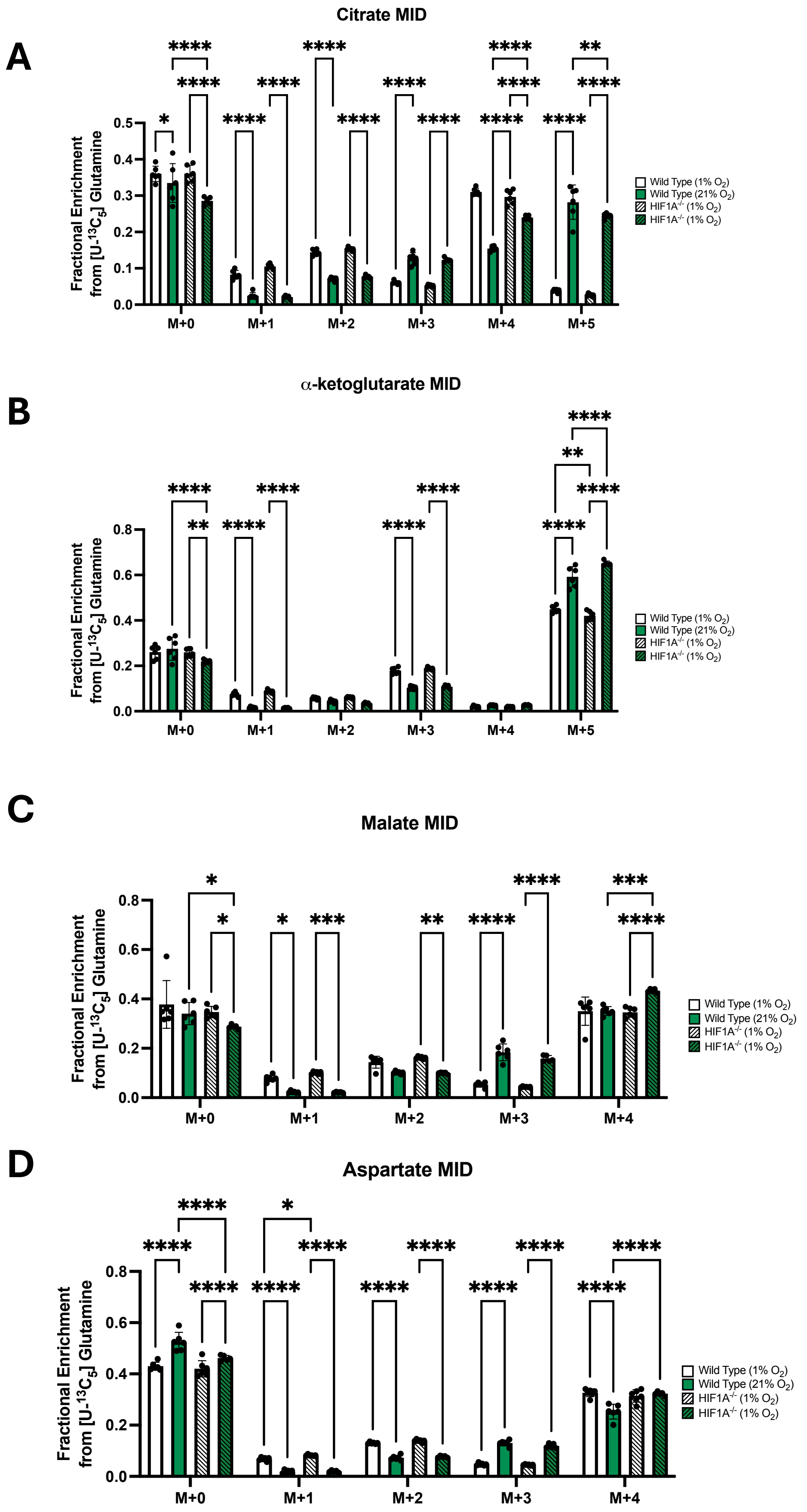

Supp. Figure 5: Isotopologues distributions for (A) citrate, (B) α-ketoglutarate, (C) malate, and (D) aspartate, from [U-$^{13}C_5$] glutamine in wild type and HIF1A$^{-/-}$ cells under normoxic (21% O_2) and hypoxic (1% O_2) conditions (24 h). Data is presented as mean ± SD for n = 6 independent experiments. Statistical analyses were performed using a two-way ANOVA, followed by Holm-Sidak post hoc test. ∗ $p < 0.05$, ∗∗ $p < 0.01$, and ∗∗∗ $p < 0.001$.

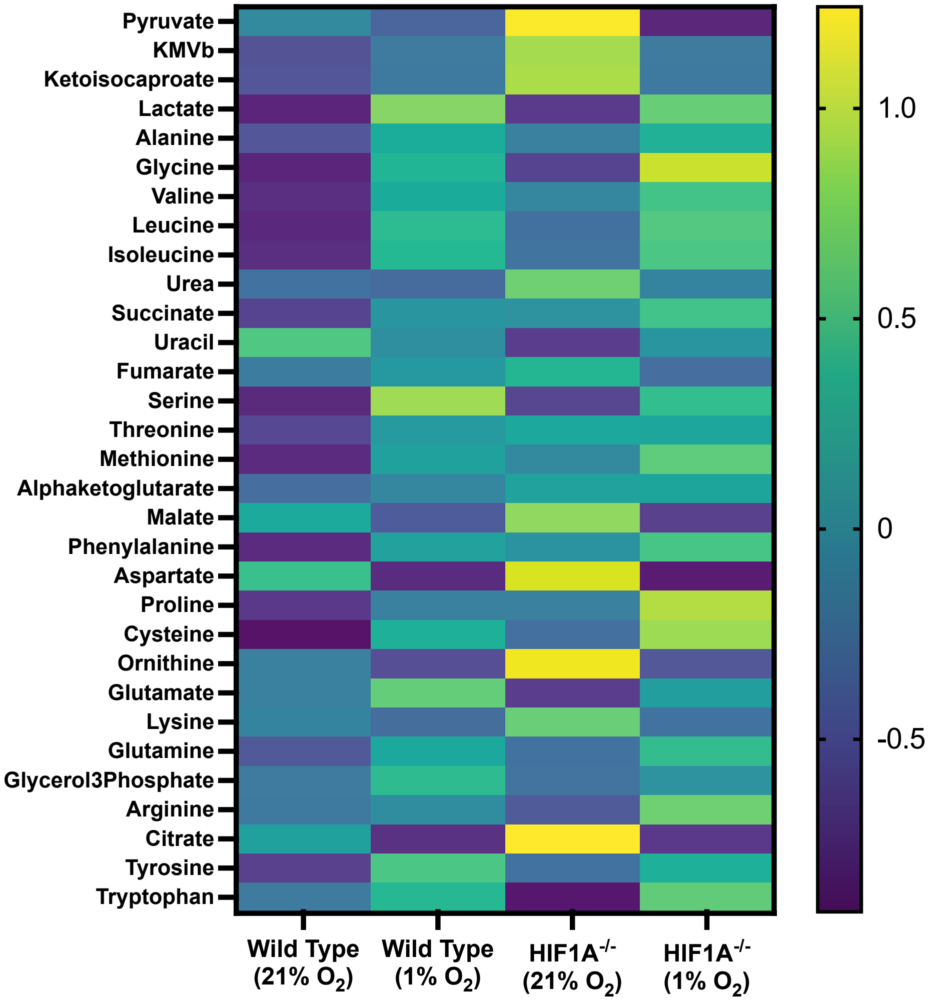

Supp Fig. 6: Heat map of relative metabolite abundances (z-score normalised) detected using a [U-$^{13}C_6$] glucose tracer in wild type and HIF1A$^{-/-}$ cells under normoxic (21% O_2) and hypoxic (1% O_2) conditions (24 h). Metabolite abundances were normalised to norvaline (internal standard) and total protein (µg) prior to z-score calculation. Each rows represent a single metabolite, and each column represents the mean abundance across 6 biological replicates for the indicated condition. Colours reflect relative metabolite abundance after z-score normalisation, with yellow indicating higher and blue indicating lower metabolite levels compared to the mean across all conditions.

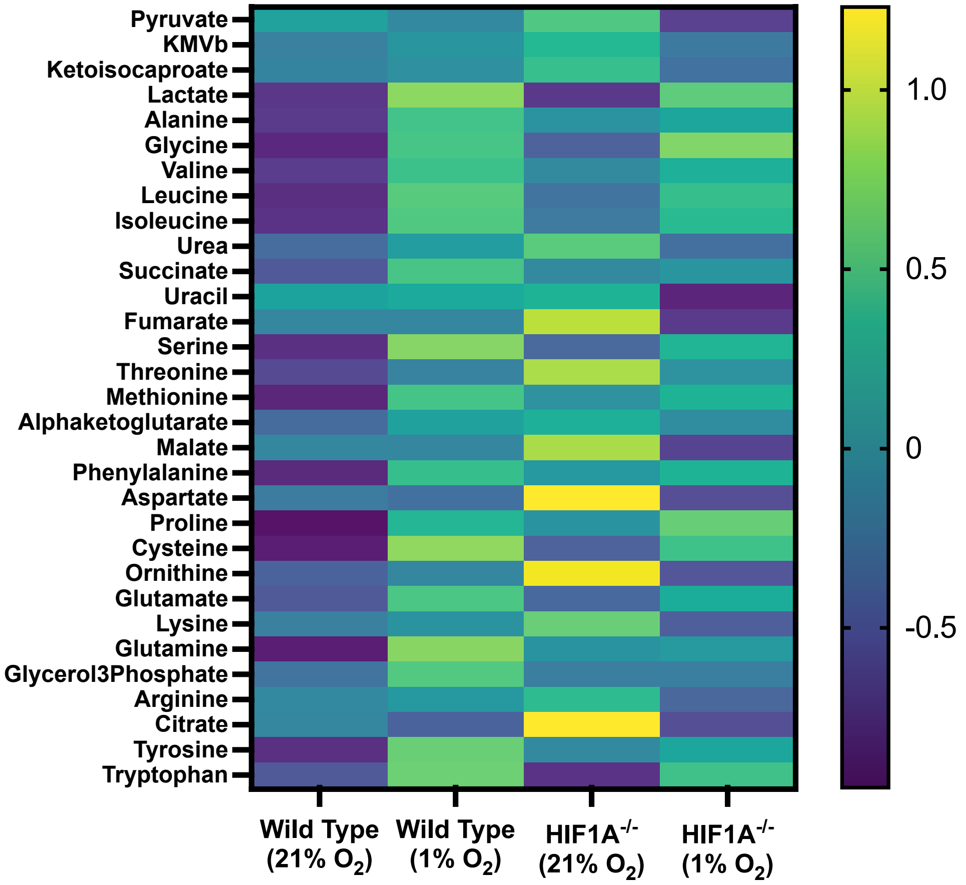

Supp Fig. 7: Heat map of relative metabolite abundances (z-score normalised) detected using a [U-$^{13}C_5$] glutamine tracer in wild type and HIF1A$^{-/-}$ Caco-2 cells under normoxic (21% O_2) and hypoxic (1% O_2) conditions (24 h). Metabolite abundances were normalised to norvaline (internal standard) and total protein (μg) prior to z-score calculation. Each rows represent a single metabolite, and each column represents the mean abundance across 6 biological replicates for the indicated condition. Colours reflect relative metabolite abundance after z-score normalisation, with yellow indicating higher and blue indicating lower metabolite levels compared to the mean across all conditions.
